# Severe megaloblastic anemia in a patient with advanced lung adenocarcinoma during treatment with erlotinib: a case report and literature review

**DOI:** 10.1186/s12890-024-02935-9

**Published:** 2024-03-06

**Authors:** Xin Yan, Jingxian Kong, Jiacheng Wang, Caixia Wang, Hongchang Shen

**Affiliations:** 1 Department of Oncology, Shandong Provincial Hospital Affiliated to Shandong First Medical University, Jinan, Shandong Province China; 2 Department of Gastroenterological surgery, Shanxian Central Hospital, Heze, Shandong Province China

**Keywords:** Megaloblastic anemia, Lung adenocarcinoma, Erlotinib

## Abstract

**Background:**

Erlotinib is a first-generation, tyrosine kinase inhibitor of the epidermal growth factor receptor (EGFR-TKI) used for the treatment patients with NSCLC. Erlotinib is considered as a safe and effective treatment option, with generally good tolerance. Diarrhea and rash are the most common side effects, and more rare side effects appear in long-term real-world applications. Severe erlotinib related megaloblastic anemia is rare and remains unreported. This is the first case report of severe megaloblastic anemia in a patient with advanced lung adenocarcinoma with an EGFR L858R mutation treated with erlotinib. In this report, the clinical manifestations, diagnosis and treatment of erlotinib related severe megaloblastic anemia are described, and the possible pathogenesis and related treatment options are discussed.

**Case description:**

Herein, we present a 57- year-old non-smoking female diagnosed with metastatic lung adenocarcinoma harboring an EGFR L858R mutation, who had received erlotinib as the first-line therapy. After 44 weeks of treatment, the patient developed severe anemia. Anemia was manifested as megaloblastic anemia with elevated mean corpuscular volume and mean corpuscular hemoglobin. The total vitamin B12 level was below the detection limit of 50.00 pg /mL. Bone marrow smear suggested megaloblastic anemia. Her hematologic parameters were markedly recovered following the withdrawal of erlotinib and vitamin B12 supplement. As a result, the patient was diagnosed with erlotinib-associated megaloblastic anemia.

**Conclusions:**

This is the first case of severe megaloblastic anemia reported with erlotinib. Few of these hematologic adverse effects have been observed in studies on erlotinib, this case report highlights this possibility for long-term erlotinib administration. Close clinical and blood monitoring is recommended for patients receiving long-term TKI therapy.

## Introduction

Approximately 350 individuals die from lung cancer daily, and lung cancer remains.

the first leading cause of cancer-related deaths [1]. Nearly half of the Asian patient with non-small cell lung cancer (NSCLC) patients carry epidermal growth factor receptor (EGFR) mutations [2]. Moreover, erlotinib has been shown to be effective in improving progression-free survival in non-small cell lung cancer patients with EGFR mutation [3]. Although osimertinib, a third-generation TKI, has shown encouraging efficacy in controlling brain metastases and the T790m mutation, its high price remains unaffordable some poor families in Asia. Therefore, many patients with lung adenocarcinoma patients choose first-generation TKI as the first-line treatment, and decide whether to use third-generation TKI after disease progression by genetic testing again. Futhermore, the most common erlotinib -associated adverse events include rash, anorexia, nausea, fatigue, vomiting and ocular toxic effect [3, 4]. A previous clinical study has reported an incidence of anemia related to erlotinib at 2-13%, and the incidence of grade 3 or above anemia at less than 1% [4–6].

Anemia is a pathological condition in which the volume of red blood cells in the systemic circulating blood is lower than normal. Patients with cancer, particularly those undergoing chemotherapy, often suffer from anemia, a serious and common condition. Concurrently, treating anemia is an important part of treatment for patients with cancer because of its potentially harmful effects on performance status, quality of life, and treatment outcomes [7]. Anemia can lead to fatigue, a condition that adversely affects the functional status of patients with cancer and places a considerable burden on them and their families. However, anemia remains frequently overlooked and undertreated [8]. Megaloblastic anemia is a unique type of anemia characterized by typical morphological changes of giant erythrocytes and red cell precursors [9]. To date, erlotinib-associated megaloblastic anemia remains unreported. Herein, we describe a rare case of megaloblastic anemia in a patient with lung adenocarcinoma harboring EGFR mutations after erlotinib treatment.

## Case presentation

**Fig. 1:**
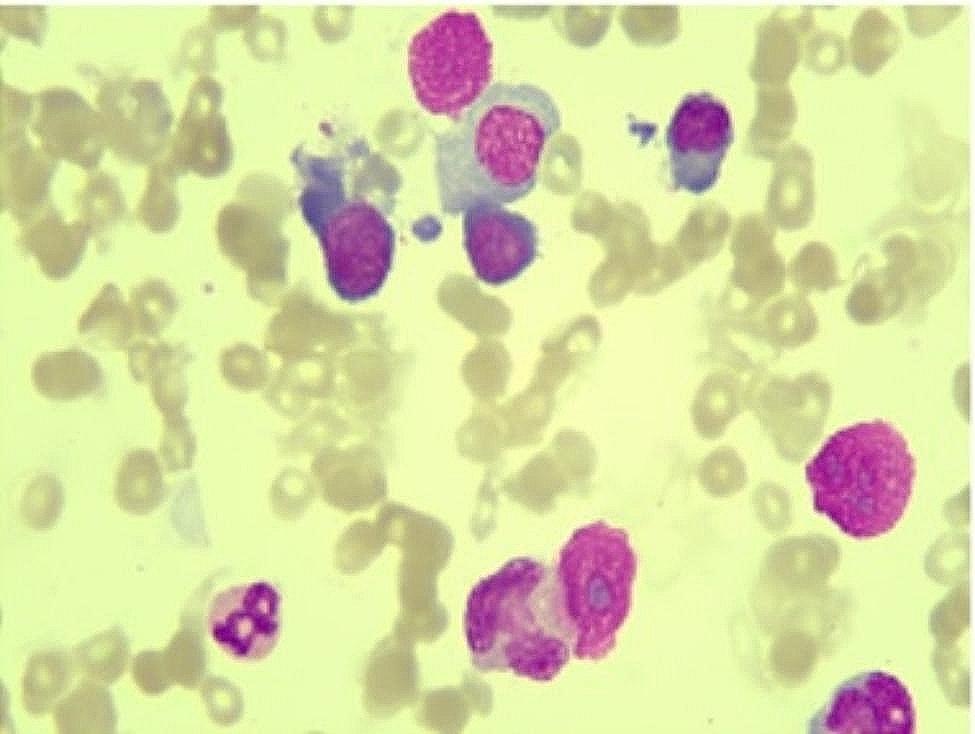
Bone marrow aspirate smears showing hypercellularity, erythroid hyperplasia, small dysplastic megakaryocytes, Howell-Jolly’s body and petal nuclear erythrocytes

A 57-year-old non-smoker woman with stage IV metastatic NSCLC with mediastinal lymph node and bone metastases (T1N2M1) harbored the activating L858R mutation of EGFR. There was no relevant gastric atrophy or pulmonary diseases information in her medical history. She had no family history of stomach disease or cancer. Her blood results were normal before treatment (Table 1). The patient was treated with oral erlotinib at a dose of 150 mg daily. After 44 weeks of erlotinib treatment, she was readmitted to the hospital with general weakness. A computed tomography scan revealed marked significant tumor enlargement. Additionally,tumor response was assessed as progressive disease according to RECIST 1.1 guidelines [10]. Hematological exams revealed severe pancytopenia (Table 1). Anemia was manifested as megaloblastic anemia with elevated MCV and MCH (see Table 1). The (total) vitamin B12 level was below the detection limit of 50.00 pg /mL [normal range180-914], but the folic acid, ferritin level and intrinsic factor antibody levels were normal. Bone marrow smear suggested megaloblastic anemia (Fig. 1). She had.

**Table 1:** Hematologic parameters of the patient

Weeks after the initiation of Erlotinib	White Blood Count, 10^9^ Cells/L	Hemoglobin level, g/L	Mean Corpuscular Volume, fl.	Mean Corpuscular Hemoglobin Concentration, pg	Absolute Neutrophil Count, 10^6^ Cells/L
0	5.15	118	92.8	316	3.05
44	1.79	45	127.3	41.1	0.9
45	11.03	70	114.5	35.6	8.34
49	9.04	97	86.3	30	7.74

been taking codeine and diclofenac sodium at least 3 months before the starting erlotinib. Additionally, she ate a balanced diet and did not consume alcoholic beverages. At the same time, she had no personal or family of gastric atrophy disease.

She did not suffer from anemia before the oral administration of erlotinib. In the absence of other causes, a diagnosis of erlotinib-induced severe megaloblastic anemia was decided. She immediately discontinued erlotinib and was treated with vitamin B12 supplementation, granulocyte colony-stimulating factor and red cell and platelet transfusions. After 1 week of treatment, the hematologic parameters got better during the hospitalization (Table 1). She received intramuscular vitamin B12 1 mg once a week outside the hospital. After 4 weeks of treatment, her hematologic parameters and weakness had markedly recovered (Table 1). The vitamin B12 level (492 pg /mL) returned to normal. She underwent a repeat biopsy of progressive tumor tissue and genetic testing, which did not identify a secondary EGFR T790M mutation, and then she was treated with carboplatin and pemetrexed. In the subsequent chemotherapy treatment, she did not develop megaloblastic anemia.

All procedures in this study were conducted in accordance with the ethical standards of the institutional and/or national research committees and the Declaration of Helsinki, as revised in 2013.Informed consent was obtained from the patients for this study, and they consented to any clinical information being published.

## Discussion

The diagnosis of megaloblastic anemia can be confirmed based on characteristic morphology and laboratory findings. Though anemia is the common feature, megaloblastic anemia could present with pancytopenia as well [11].

Megaloblastic anemia is mainly caused by vitamin B12 deficiency or folate deficiency. The causes of vitamin B12 include autoimmune gastritis, inflammatory bowel disease, postoperative surgery, vegetarians, and drug effects [9]. 

This patient developed severe pancytopenia after oral erlotinib, and megaloblastic anemia was confirmed by a series of laboratory investigations. She occasionally took oral codeine and diclofenac sodium tablets, which have been previously been reported to cause serious hematologic toxicity, and the patient used these drugs 3 months before taking erlotinib. She developed a vitamin B12 deficiency, but the endogenous factor antibody, folic acid and ferritin levels were normal. Besides, she eats a balanced diet and is not a vegetarian. Furthermore, the anemia was corrected after erlotinib discontinuation. Therefore, we considered erlotinib to be the most likely cause of severe megaloblastic anemia.

Drugs can cause megaloblastic anemia by impairing cellular utilization or the use of folate or vitamin B12 through a variety of mechanisms. These possible causes include interference with folate or vitamin B12 absorption, plasma transport, or delivery, competition for reductases, etc [12]. Many antineoplastic drugs can cause megaloblastic anemia, such as thioguanine, gemcitabine and pemetrexed, etc [13]. However, megaloblastic anemia caused by EGFR TKIs are very rare. To date, the cases of megaloblastic anemia caused by other TKIs are also very rare, only sunitinib-induced megaloblastic anemia have been published [14]. To our knowledge, this is the first case of severe megaloblastic anemia reported with erlotinib. The mechanism of vitamin B12 deficiency and megaloblastic anemia caused by erlotinib remains unclear and requires further explorations. In addition, we suggest that TKIs, including erlotinib, may contribute to megaloblastic anemia caused by vitamin B12 deficiency.

In conclusion, we report a rare case of megaloblastic anemia that developed in a 57-year-old woman after long-term erlotinib administration. Oncologists should be aware of the risk of severe megaloblastic anemia induced by erlotinib and monitor hematologic toxicities and vitamin B12 levels in patients.

## Data Availability

All data and materials in this study are included in this published article.
